# Technology: Saving and Enriching Life During COVID-19

**DOI:** 10.3389/fpsyg.2021.647681

**Published:** 2021-03-29

**Authors:** Shubhra Sinha, Ankita Verma, Priyanka Tiwari

**Affiliations:** ^1^Department of Psychology, Vasant Kanya Mahavidyalaya, Varanasi, India; ^2^Department of Humanities and Social Sciences, Indian Institute of Technology Roorkee, Roorkee, India

**Keywords:** social network site, COVID-19, elderly, senior citizen, loneliness, digital payment

## Abstract

The pandemic of COVID-19 has arrested the life of 7.8 million people living on this earth. However, some people are more vulnerable to the risk of this deadly virus. The frailty of senior citizens put them at the top of this list. The past 6 months have not only presented a threat to their physical health but to mental health also. Although lockdown was necessary to check the spread of the coronavirus it culminated in an exponential rise in the problems of loneliness, anxiety, fear, helplessness, and depression. The present paper reviews the role of social networking sites, apps, and other digital platforms in saving and enriching the lives of the elderly, especially those who spent the lockdown alone and were devoid of a regular support system due to unavailability of transport and administrative restrictions on the movement of people. It also analyzes the efficiency of the virtual world in reducing their anxiety of being alone by connecting them with others and also make them feel empowered. The review is based on the online data collected about the insurgence in the percentage of elderly people using such platforms, recent studies analyzing the effects of the COVID 19 pandemic on senior citizens. Besides this personal telephonic discussions were conducted with some elderly people who spent their lockdown alone in their homes. The study was primarily focused on three objectives. Firstly it attempts to understand the ways in which senior citizens made use of social networking sites and various digital platforms for managing life better. Secondly, it analyses the process of adopting technology, and finally, it examined the width and depth of the impact technology created in their life and also the permanence of this change. The analysis clearly suggests an increase in the digital life of elderly people. The process moved in distinct stages from utter confusion to relative ease in using technology, thereby significantly reducing the loneliness, and bringing relatively stable change in the way they lead their life.

## Introduction

The pandemic of COVID-19 has arrested the lives of 7.8 million people living on this earth. After discovering the first infected case in Wuhan, China, in December 2019, COVID-19 has spread beyond China, Asia, and the rest of the world, raising an unprecedented public health problem.

The World Health Organisation (WHO), on March 11, 2020, announced COVID-19 as a pandemic, and as of March 24, 2020, more than 3.5 lakhs cases were confirmed, and more than 14,000 deaths were registered, affecting 190 countries worldwide (WHO website dated March 24, 2020, at 9:00 p.m. Indian standard time) (Coronavirus, 2019). These figures have exponentially increased to about 27.19 lakhs cases with about 1.9 lakhs deaths in 1 month time (WHO website date April 25, 2020, at 05:30 p.m. Indian standard time) (Holmes, 1999).

In response to “flatten the curve” and curb the community spread, In India, a national level “lockdown” was declared starting from midnight of March 25, 2020, initially for 21 days, which was later extended up to May 3, 2020 (The Lancet, 2020). “Lockdown” is an emergency protocol that restricts the public from traveling from one location to another. However, complete lockdown ensures that people remain where they are presently, and the government will permit no entry/exit motions. Although lockdown proves to be a powerful and effective technique to counter the increasing spread of the highly contagious COVID-19 virus, the key side effect of lockdown is that it exacerbated psychological well-being among the citizens. The popular slogan of “social distancing,” which refers to physical distancing among individuals in social settings, gradually ushered people toward loneliness. Social isolation and quarantine, the primary treatment measures adopted, increased the psychological distress among patients and their caregiver manifolds. This results in significant mental health problems ranging from anxiety, fear, sense of loneliness, depressive symptoms, anger, sleep disturbances, etc. Similar findings were reported on SARS (Severe Acute Respiratory distress Syndrome) patients when they were kept in isolation (Reynolds et al., 2008).

However, the level of risk and distress people suffered from was of varying degrees. According to a survey by the US Centers for Disease Control and Prevention (CDC) in March 2020, more than 80% of deaths have been reported in patients over 65 years of age, indicating vulnerability of the elderly to the virus (Bialek et al., 2020; Li et al., 2020). The picture is largely the same in India also. According to the WHO data elderly (above 60) account for 51% of the 1,48,153 deaths in India (Guitton, 2020). Because of this, it is most likely that others might avoid meeting them, which further aggravates the consequences of social distancing. Santini et al. (2020) further verified this fact; they recently demonstrated that social disconnection puts older adults at an increased risk of depression and anxiety.

From a technological perspective, the COVID-19 pandemic has provoked massive, immediate switching to online platforms. With unprecedented changes in population use of digital technologies and media (Guitton, 2020), the digital platform’s status changes from an amenity to a priority; not only are they the primary means of accessing information and resources, but they are also one of the only surviving routes for physical, educational and leisure activities and also for social interactions. This change is observed in all sections of the population, especially people who are in their 60s. In India, 25–30% of senior citizens are now active on the internet as compared to the 6–8% active users 6 months ago (Statista). This is an exponential rise in the number of users in this age group. A lot has been said and written to describe the increasing digital literacy among the elderly. However, a systematic and comprehensive study examining the interactive influence of the pandemic and technology on the life of the elderly is still needed. Precisely it would include the analysis of the functional utility of technology for neutralizing the physical and psycho-social risk of infection on the one hand and the compelling effect of a pandemic that broke the techno-resistance in the mind of people and ushered them to new normal away from their conventional way of doing things on the other. Therefore, a small qualitative study was designed, which primarily focused on three objectives. Firstly, it attempted to understand the ways in which senior citizens made use of social networking sites and various digital platforms for managing life better. Secondly, it analyzed the process of adapting to technology. Finally, it examined the width and depth of the impact technology created in their life and the permanence of this change.

## Materials and Methods

Suitable participants were identified through convenient sampling and contacted over the phone. The objectives of the study were explained to them and their consent was taken. Proper appointment was fixed for the interview at a suitable time when they can easily spare 30 min for the interview. Special attention was given to rapport building before actual questions were asked. Interview was conducted on a video call connected through whatsapp and their responses were noted verbatim by the researcher. It started with simple questions about themselves and their family like their name, age, and residence to warm them up and also to make them comfortable before actual questions were asked. Familiarity with and use of technology is not a tabooed topic to talk about so it was not difficult at all to get responses. In fact, respondents enjoyed discussing various issues during the interview, which shows their positive inclination toward the interview. The scripts were later on analyzed for the basic issues raised in the study.

### Sample

In view of the objectives elderly people living all alone or with husband/wife at least for 5 years were identified. While selecting the sample it was ensured that (a) they have spent the whole lockdown away from their children and other members of their extended family, (b) they had access to smart-phones and laptops/desktops, which are the essential tools for entering into the virtual world. Convenience sampling method was used and 20 participants were included in the sample. Males (*n* = 10) and females (*n* = 10) were equally represented in the sample. Age ranged from 58 to 65 years. As the sample was selected through convenience sampling, especial care was taken to ensure the external validity of the sample. Participants were selected from five different states (i.e., Delhi, Rajasthan, Kerala, Uttar Pradesh, and Bihar) and 11 different cities (i.e., Varanasi, Mirzapur, Deoria, Ballia, Patna, Kushinagar, Gorakhpur, Trivendram, Jodhpur, Ghazaibad, and Delhi) of the country.

Participants came from diverse professional backgrounds. Among the female participants six were home-makers, two were Post Graduate teachers in government schools, one was working in the private sector and one retired teacher (govt. School) was there. Among male participants five were retired from various government offices and five were in jobs. Out of these five, one was Sub-Inspector Police, one was a manager in Life Insurance Corporation, one was a contractor, and two were bank employees. When asked about their SES they all reported their monthly income between 40,000 and 100,000 INR.

Information about current health status revealed that 12 participants were suffering from problems like high blood pressure, diabetes, thyroid and goiter but they were functional enough to do their daily job. None of them were suffering from any serious or terminal illness. The participants were never found infected with COVID-19 during the lockdown.

#### Instrument

This is a small qualitative study based on narrative approach where narratives were generated through a structured in-depth interview having twenty questions to understand the importance and use of electronic devices, digital payment methods and social networking sites in the life of senior citizens during the lockdown. Interview also focused on the way various social networking sites and digital payment apps helped senior citizens to manage their lives during this lockdown.

#### The Interview-Schedule

An interview-schedule was developed to get a deeper insight into the impact of COVID-19 on the lives of elderly and its proper management with the use of technology. Core issues addressed by the interview were (a) familiarity with technology, (b) reluctance to technology, (c) difficulties in initiation, (d) functional utility of technology, and (e) long-term capacity building. Initially thirty questions were written. Thereafter based on elaborated discussions twenty questions were retained for their clarity and scope to elicit responses. The schedule contains twenty questions. The first section has five questions devoted to assessing their relationship with children. For example, how frequently do your children visit you? (Q. no. 3) and how much emotional support did you get from your children during the lockdown? (Q. no 5). Next section also had five questions related to the familiarity and utility of technology in daily life before COVID-19. For example, whether technology was a part of your life prior to COVID-19? (Q. no. 6). Have you used any digital payment mode prior to COVID-19? (Q. no. 9). Section three was devoted to understanding the reasons of resistance to technology and difficulties faced by the participants when they started using it. This section contained two questions viz., why didn’t you use social networking sites/online shopping/payment before? (Q. no. 11) and when you started using these online platforms what were the difficulties or challenges that you faced? (Q. no. 12). Section four had five questions that gather information about the various usage of these online platforms during the lockdown. Sample questions are which social networking sites have you used to connect to your family? (Q. no. 13) or how these online platforms helped you reduce your anxiety and loneliness during lockdown (Q. no. 16). The last section had three questions. Q. 18 asked participants to describe their journey of learning and using technology. Q. 19 will you continue using these online mechanisms even after lockdown. Unlike the other questions, Q. 20 asked for direct comparison of seven important aspects of participants’ lives pre and post lockdown on a five-point scale, where 1 represented minimum and 5 represented maximum. These were (i) friendliness of people around, (ii) confidence in using technology, (iii) connectedness with people around, (iv) experience of self-sufficiency, (v) level of self-esteem, (vi) level of mental alertness, and(vii) overall mental health.

Questionnaires were initially prepared in Hindi but considering the wider regional coverage in sample selection the questions were translated in English as well using the translation-back-translation method for the convenience of respondents.

## Results

Content analysis of the transcripts started with examining the quality of participants’ relationship with their children. This is an important factor determining the level of loneliness in elderly. Responses revealed that most of the participants were living alone for 5 years or less than that. Couples are living alone because their children are out for job/studies or are settled at a distant place after marriage. Participants reported regular visits of children before lockdown. The frequency of visits varies from once a week to once in 6 months depending upon the distance between the places they reside. During lockdown the children were continuously in touch with their parents over phone, WhatsApp, and video call. No history of family conflict or broken relationships was reported by anyone of them. So, living alone is due to situational constraints not because of any family dispute or broken relationships.

### Familiarity With and Reluctance to the Technology

Familiarity with digital platforms or technology refers to the knowledge of availability and functional utility of these things and does not necessarily include first hand use. Reluctance to technology refers to the reservations of participants for using communication technology, e-commerce sites, digital payment modes and social networking sites over other conventional methods of doing things.

While examining the familiarity of participants with the digital world that there were two levels of this familiarity. First was the familiarity with the tools and second was the familiarity with the software. Participants started their narration by saying that they have smart phones and laptops. When asked about their usage the most common response was that they use smartphones to make voice calls, video calls, and send WhatsApp messages. Six of them had their Facebook accounts also but were not very active on it except one who was the Sub-Inspector in the police department. So, there was a clear preference for communication technology over e-commerce technology. Participants were also using professional softwares relevant to their jobs. For example, the banker was working with the banking software, the LIC agent was using his office software. However, there was no such techno-dependency of other participants.

Incidental use of online shopping and digital payments was reported. when asked about the ease of online shopping, which saves time and energy, participants said that local shopkeepers also give doorstep delivery. “We order routine stuff (especially grocery, fruits, vegetables, milk, bakery items) over phone and shops would deliver and we can pay them in cash.”

#### Reluctance to Technology

The reasons for this limited use of various digital/online tools were (a) face to face interaction was preferred over the virtual mode, (b) fear that is not safe; financial transaction is risky even sharing information on Facebook has a risk of unwanted use of personal details (c) difficult to understand, icons were confusing (d) could not find enough time to learn, (e) didn’t feel a need to use technology, (f) children would do the things for us, as they don’t find time to teach these things, (g) online shopping is difficult as we cannot try product before buying, the color, texture, and quality can be judged better if you see it. (h) what if the company doesn’t pick up the product we want to return, (h) it takes a lot of time to get the product online whereas we can directly purchase it from the market. One of the participants was not using technology at all. So, it can be said that these people were familiar with the available online options but these options were not much preferred over the offline options.

### Difficulty in Initiation

All of them have started using these things during lockdown. however, the process seemed to be fairly difficult to initiate. Participants reported that it was confusing and difficult to understand. There is an unknown fear of things going wrong, instructions and options written in English were not clear to them. They didn’t know how to change the language. Operating a smart phone or laptop was not so simple for them. The situation was even more difficult for those in job because they had to accommodate the professional requirements that too with the newly learned technology. There was no one to help them learn these things. Children instructed them over phone, which was not enough. Some of them tried to see YouTube videos to learn using technology.

#### Functional Utility During Lockdown

People used online platforms for (i) making video calls and talking to friends and relatives, (ii) buying things/medicines online, (iii) financial transactions, (iv) official meetings, (v) learning new things on YouTube for managing one’s leisure time. Facebook, WhatsApp, and YouTube are the most used sites. Some people have used zoom meeting app also. Almost all payment methods have been explored including Google pay, UPI (Unified Payment Interface), net banking, Paytm, Phone pe etc. participants used Amazon, Myntra, and Flipkart to buy things but it is largely combined with offline shopping especially for Grocery, fruits, vegetables, milk, and milk products. Only two participants said that they used online shopping to buy these kinds of stuff. Others preferred to buy these things offline. Either they go to the market or they ordered it on phone and the shopkeepers delivered it or there were some arrangements made by the local governing bodies to ensure proper distribution of these things in every locality. Participants reported that they had not required online medical consultation apps. If any such thing was required, they called their doctors and managed to get the prescription. Instead, they used online platforms to buy medicines other than ordering them on phone. They also said that SNS and other online platforms helped to overcome anxiety and loneliness as it was a nice way to spend time and learn something new and fruitful. Online shopping was an interesting experience. We could talk to family members and relatives over a video call, which reduced anxiety to a great extent as we could see them and discuss things with them.

#### Long Term Capacity Building

All the participants reported that they will continue using technology even after COVID is completely over. They all described it as a journey of learning new things. They say that they are still in the learning phase. It was full of knowledge but difficult too. A direct pre- and post-lockdown comparison revealed that (a) people perceive people around them as less friendly than before COVID, (b) they felt more confident in using technology, (c) there is a slight increase in social connectedness, self-sufficiency, and self-esteem after lockdown and (d) the level of mental alertness and mental health was almost the same before and after lockdown.

## Discussion

The present study was taken up to understand how SNS, digital payment modes, online shopping, and various apps help senior citizens manage their lives well during the lockdown.

The first step toward understanding the matter was the assessment of the participants’ awareness of the availability and functions of these things and then to have an idea of the use of technology in daily life. Here the familiarity with technology and reluctance toward its use both are high. This means the unavailability of gadgets and the internet is not the only reason for its limited use by the elderly; psychological factors are also important. The apprehension of things being messed up or becoming a victim of online fraud while using a digital payment system or perceived inability to learn something new or the mindset that the conventional methods are better than the digital ones all are psychological factors that need to be addressed before elderly can become active users of technology. Similar findings were reported by studies carried out in the US and other European countries also (Yuan et al., 2016). These studies (e.g., Marquié et al., 2002; Selwyn et al., 2003) identify several non-cognitive factors such as lack of interest, perceived redundancy of technology, fear of computerization, negative attitude toward spread of technology, and lack of confidence as potential deterrents for using technology.

However, the sudden lockdown and the fear of contamination by the deadly CORONAvirus put the elderly under strict social isolation (Chen, 2020; Jordan et al., 2020; Niu et al., 2020). The fact that Information and Communication Technology was the only way to maintain contact with the outer world motivated them to practice what they knew. Examining the pattern of use reported by the participants clearly showed their inclination for communication technology. The technology was most frequently used to contact children and other family members living in distant places. Video calls took over the audio calls, and various online meeting apps like Google Duo and Zoom were also used for making family calls, which were like family reunion events during the lockdown. These platforms were also used for professional meetings with colleagues by those who were on the job. The two teachers in our sample used these platforms for taking classes in the new normal. The next preferred online service was digital payment. The participants explored different payment modes like G pay, Paytm, PhonePe, and other banking apps. For shopping of utility items, the trend was mixed. Participants preferred shopping the groceries and milk products from local shopkeepers over the phone, whereas other items were ordered on e-commerce sites. Amazon, Flipkart, Myntra, Snapdeal were the sites most commonly cited by the respondents. Unquestionably, the Information and Communication Technology was the biggest savior during the COVID-19 pandemic. However, it was not a smooth shift to the new normal from the conventional methods for most of us, especially for the elderly who had their reservations about technology use.

While talking of the difficulties in learning/using technology, the participants’ most common problem was the lack of a proper support system. Children and other family members usually lack time and patience to match the learning pace of the elderly. Online aids are not easy to explore, and they also fail to accommodate the special needs of the elderly (Berkowsky et al., 2013). However, the extraordinary circumstances of the pandemic played a significant role in sensitizing people about this issue. Children themselves became the biggest motivator and instructed their parents over the phone about how to use a particular application. Number of specially tailored Youtube videos demonstrating the use of these things also increased manifolds.

The use of online platforms compensated for the face-to-face interaction with family and friends on the one hand and also enhanced their self-sufficiency, confidence, and self-esteem. The mechanism was also fascinating. After a certain age, when the elderly do not remain very open to new things and become confined to the conventional ways of doing things, they somehow become dependent on their children for (i) information, (ii) decision-making, and (iii) execution of that decision. The use of online services made the state-of-the-art information available at their doorstep. It simplified the exchange of information and facilitated discussion about a service or product with similar others, thereby helping them make proper decisions. The availability of various online shopping platforms, digital payment modes, and online apps like Urbanclap, on which they can book various services online, has made life easy. Availability of medical consultation apps like Practo that gives a package of consultation, diagnostic testing, and allied services like physiotherapy has reassured them easy access to all required facilities without dwelling into the hassles of going to the hospital everyday health problems. Mynatt and Rogers (2001) also highlight the importance of using technology in the “functional independence” and “independent living” of the elderly in their houses.

Few participants who were very new to the online world reported that they have started using SNS and online services, but they do not do it themselves; instead, their husbands have learned to use these things, and they do it for them. Maybe getting good experience and watching others using technology is the first step for overcoming resistance to it. Responses suggest that in the future, they would like to give it a try.

Findings reveal vivid uses of the technology and reaffirm the virtual world’s efficiency to manage real-life crises. It gives very useful lessons to facilitate the diffusion of technology in the larger society, especially in those pockets with their own specific needs like remote rural areas, young children, females, and senior citizens. This also allows assessing the extent to which hiatus of the real world can be compensated by the virtual world, at least to some extent. It was a practical demonstration of the saying “there is no age to learn.” For example, Facebook can help get help from people around in the hour of need when you stay away from your family. SNS gives an excellent method of talking and expressing oneself, which is a healing mechanism in itself. This is especially true for the elderly population, who often suffer from being heard of. This is substantiated because participants were happily talking to their distant relatives who were not in touch for a long time.

Moreover, this learning exercise has the immediate benefit of adding knowledge and skills and has a long-term benefit of keeping their brain active, reducing the risk of dementia and other age-related cognitive deterioration. This fact is reiterated on many occasions by many institutes working with the mental health of the elderly. The National Institute of Ageing, United States, emphasizes the importance of continuous stimulation of the brain through various sensory modalities, and learning something new is the best way to do that.

Overall, it can be said that technology has a synergistic effect on human adaptability. It has tremendous scope for crisis management. Future research must focus on simplification and effective dissemination of online tools and resources to develop an efficient crisis management system.

## Data Availability Statement

The original contributions presented in the study are included in the article/Supplementary Material, further inquiries can be directed to the corresponding author/s.

## Ethics Statement

Ethical review and approval was not required for the study on human participants in accordance with the local legislation and institutional requirements. The patients/participants provided their written informed consent to participate in this study.

## Author Contributions

SS and AV has equally contributed and were supported by PT. All authors contributed to the article and approved the submitted version.

## Conflict of Interest

The authors declare that the research was conducted in the absence of any commercial or financial relationships that could be construed as a potential conflict of interest.
