# Supplementary material for: Technology: Saving and Enriching Life During COVID-19
Source: Front Psychol. 2021 Mar 29;12:647681. doi: 10.3389/fpsyg.2021.647681 (PMC8040793; doi:10.3389/fpsyg.2021.647681)
Supplement: Supplementary file 1 [file Data_Sheet_1.pdf]

# Technology and COVID-19

This is a brief questionnaire to share your experiences with technology and online exposure during the Lockdown. The main aim of this questionnaire is to understand the problems you might have faced due to sudden and unprecedented changes in your life and the innovative approaches you would have applied to address these issues using technology and social networking sites.

Your effort to be accurate and precise while answering is highly appreciated. Data collected from you is highly confidential and will only be used for Research purpose. Research team is highly grateful for your time, effort and participation.

*Note-This questionnaire is divided into three sections, Demographic Section, Subjective question and answer Section and Multiple-choice Question Section.*

## Demographic Details

Name of the Participant-

Age of the Participant-

Gender-

Current Residing Location-

Marital Status-

Lives all alone or with Husband/wife-

Your Profession-

Current working Status-

Please list the Chronic Health Problems you are suffering from (if any)-

**Subjective Questions**

**Please answer the following questions**

1 For how long are you living alone?
2 Where are your children?
3 How frequently do your children visit you?
4 How much supportive and caring is your family members?
5 How much emotional support do you receive from your family members during the Lockdown?
6 Was technology a part of your life before COVID-19?
7 Have you ever used any online platform/payment mode of shopping/ financial transaction before COVID-19 on a regular basis?
8 (a) If Yes, what are those?
9 (b) If not, have you started using these during COVID period especially lockdown?
10 Which social networking site/digital payment modes/ entertainment sites have you started using to connect to your family?
11 Why didn't you use technology ever before?
12 What were the challenges and difficulties you faced when you started using technology?
13 How did you manage to get the grocery, fruits, vegetables, and medicines in the lockdown period?
14 How online services facilitated the regular medical care consultations during this period?
15 Who helped you the most in learning the use of technology?
16 How did it helped reducing your anxiety and loneliness during loneliness?
17 How did it help you to be connected with your friends and family?
18 Overall, how do you describe your journey from not using technology to learning, practicing, and mastering technology?
19 Will you continue using these technologies after complete unlock also?

**20. In continuation with the questions asked above following are some multiple-choice questions. Please rate the alternative nearest to your pre-and post-pandemic experience.**

Pre-Lockdown Post-Lockdown

| S. No. | 1 | 2 | 3 | 4 | 5 | | 1 | 2 | 3 | 4 | 5 |
|---|---|---|---|---|---|---|---|---|---|---|---|
| 1 | | | | | | Friendliness of people around | | | | | |
| 2 | | | | | | Enhanced Confidence due to use of technology | | | | | |
| 3 | | | | | | Connectedness with people around | | | | | |
| 4 | | | | | | Experience of self-sufficiency | | | | | |
| 5 | | | | | | Level of Self- esteem | | | | | |
| 6 | | | | | | Level of Mental Alertness | | | | | |
| 7 | | | | | | Overall Mental Health | | | | | |

Please Note

1 = Strongly Disagree

2 = Disagree

3 = Neutral

4 = Agree

5 = Strongly Agree
